# Potential Excess Mortality in *BRCA1/2* Mutation Carriers beyond Breast, Ovarian, Prostate, and Pancreatic Cancers, and Melanoma

**DOI:** 10.1371/journal.pone.0004812

**Published:** 2009-03-11

**Authors:** Phuong L. Mai, Nilanjan Chatterjee, Patricia Hartge, Margaret Tucker, Lawrence Brody, Jeffery P. Struewing, Sholom Wacholder

**Affiliations:** 1 Clinical Genetic Branch, National Cancer Institute, Rockville, Maryland, United States of America; 2 Biostatistics Branch, National Cancer Institute, Rockville, Maryland, United States of America; 3 Office of Director of the Biostatistics and Epidemiology Program, National Cancer Institute, Rockville, Maryland, United States of America; 4 Genetic Epidemiology Branch of the Division of Cancer Epidemiology and Genetics, National Cancer Institute, Rockville, Maryland, United States of America; 5 National Human Genome Research Institute, Rockville, Maryland, United States of America; Ohio State University Medical Center, United States of America

## Abstract

**Background:**

Although the increase in risk of developing breast, ovarian, and prostate cancer in *BRCA1* and *BRCA2* mutation carriers has been studied extensively, its impact on mortality is not well quantified. Further, possible effect of *BRCA* mutations on non-cancer mortality risk has not been examined.

**Methodology/Principal Findings:**

Using mortality data from the relatives of 5,287 genotyped participants, of whom 120 carried a *BRCA* Ashkenazi Jewish founder mutation, in a community-based study of the Ashkenazi Jewish population in the Washington D.C area, we examined the association between the three Ashkenazi *BRCA* founder mutations and risk of overall and non-cancer mortality. To examine risks beyond the established effects of these mutations, we analyzed the data excluding both deaths and follow-up times after reported diagnosis of melanoma and cancer of the breast, ovary, prostate, and pancreas. Using an extension of the kin-cohort method that accounts for informative censoring, we estimated that, in the absence of breast, ovarian, and pancreatic cancers, and melanoma, female carriers had a life expectancy that was 6.8 years lower (95% CI: 1.2–10.5) than non-carriers. In male mutation carriers, the reduction in life expectancy, in the absence of prostate and pancreatic cancers and melanoma, was 3.7 (95% CI: −0.4, 6.8) years. When deaths and follow-up times after any cancer diagnosis were excluded, the difference in life expectancy was 5.7 years for women (95% CI: −0.1, 10.4) and 3.7 years for men (95% CI: −0.4, 6.9). An overall test of association for men and women together showed a statistically significant association between *BRCA1/2* mutations and increased non-cancer mortality (p = 0.024).

**Conclusions/Significance:**

These findings suggest that there may be unknown effects of *BRCA1/2* mutations on non-neoplastic diseases that cause death at older ages.

## Introduction

Germline mutations in the *BRCA1* and *BRCA2* (*BRCA1/2*) tumor suppressor genes are highly penetrant for increased risks of breast and ovarian cancers [1]–[9]. Other cancers that have been shown to be associated with *BRCA1/2* mutations include male breast cancer, prostate cancer, pancreatic cancer, and melanoma [8], [10]–[12]. A recent population-based study from Canada suggested that *BRCA1/2* mutations were associated with a significantly increased risk of cancers overall and at sites other than breast and ovary [12].

Although the exact role of these genes on carcinogenesis is not fully understood, existing data suggest that they play a key role in DNA damage repair and the maintenance of genomic stability [13]. Very little is known about the effects of *BRCA1/2* mutations on phenotypes other than cancer. Moreover, although overall life expectancy in *BRCA1/2* mutation carriers has been estimated, using simulation models, to be decreased [14], the actual impact of having a *BRCA1/2* mutation on life expectancy is not known. A recent actuarial analysis in female *BRCA1* and *BRCA2* mutation carriers showed that mortality risk by age 70 was greater among *BRCA1* mutation carriers than among *BRCA2* mutation carriers; and that the major contributing causes of death for both *BRCA1* and *BRCA2* mutation carriers were breast cancer and ovarian cancer [15]. There has been no report to date on non-cancer mortality among *BRCA1/2* mutation carriers.

To examine the effect of *BRCA1/2* mutations on mortality apart from their known effects on cancer risk, we studied the association between mutations and mortality in the absence of cancer in a cohort of first-degree relatives (FDR) of known *BRCA1/2* mutation carriers and non-carriers. We were able to use a kin-cohort analysis that accounts for informative censoring to estimate the effect of *BRCA1/2* mutations on mortality with the effect of mortality directly related to cancer removed [16].

## Methods

### Ethics Statement

This study was conducted according to the principles expressed in the Declaration of Helsinki. Use of human subject data in this study was approved by the Special Study Institutional Review Board and Clinical Center Institutional Review Board of the National Cancer Institute, in accordance with an assurance filed with and approved by the Department of Health and Human Services. All patients provided written informed consent for the collection of samples and subsequent analysis.

We used data available from the cohort of FDR of participants in the Washington Ashkenazi Study (WAS) [6] to study the association between *BRCA1/2* mutations and mortality risk at different ages for both men and women. Details of subject recruitment methods, data collection and laboratory testing for the WAS have been reported elsewhere [6]. In brief, Ashkenazi Jewish men and women living in the Washington D.C area were recruited through public media such as posters, newspaper, and radio announcements. A total of 5,318 volunteers were enrolled over a nine-week period in 1996. All participants provided blood samples after giving written informed consent. Genotyping for the three specific Ashkenazi founder mutations in this population, 5382insC and 185delAG in *BRCA1* and 6174delT in *BRCA2*, was performed with PCR-based assays for all individuals enrolled in the WAS. Through a self-administered questionnaire, the participants also provided detailed information regarding their family history of cancer, including age at diagnosis, vital status information, year of birth and year of death, for all FDR. Thirty one participants who did not contribute any relative with known vital status were excluded from this report. Cause of death was not ascertained and could not be retrieved because the data were anonymized. First-degree relatives were categorized into parents, siblings, and children. For families in which two or more family members participated in the study, only one member was selected in the determination of carrier status for FDR; thus no relative was included more than once. If volunteers from the same family included a mutation carrier and a non-carrier, the mutation carrier was used as the proband.

We previously described the “kin-cohort” analytic approach, demonstrating how the penetrance of a disease related to a genetic mutation can be estimated from the disease history data of the relatives of genotyped subjects [17]. This method relies on the basic principle that, although the genotype of the relatives are not known, one can use Mendelian laws to estimate the expected proportions of carriers and non-carriers among the relatives of the genotyped individuals; alternatively, one can think of the cohort of relatives as having “missing” genotypes, whose distribution can be inferred via Mendelian laws from participants who were genotyped. Generally, the proportion of carriers of a rare mutation will be much higher in close relatives of a mutation carrier than in relatives of a mutation non-carrier. We can test the association between mutation and disease, as well as estimate genotype-specific absolute risk (penetrance) for the disease using the inferred genotype distribution for all relatives. Following the same principles as in our studies of cancer incidence [6], [17] and survival after diagnosis [18], we used mortality data from the relatives of the WAS participants to obtain age-specific cumulative risks and hazards (interval risk) of mortality among *BRCA1/2* mutation carriers and non-carriers.

### Mortality in the absence of *BRCA*-related cancers

We treated a diagnosis of breast, ovarian, prostate, pancreatic cancer, or melanoma as a censoring event to eliminate the established effects of *BRCA1/2* mutations on mortality risk. Unlike previous applications of kin-cohort estimation [17], [19]–[21], we could not assume that the censoring events were independent of the mutations under study because diagnosis of a *BRCA1/2* mutation-related cancer made the unknown genotype more likely to include a mutation. The analytic approach of Chatterjee and Wacholder [16] accounts for possible bias due to this dependent censoring. We estimated hazards of mortality for carriers and non-carriers in 10-year intervals and plotted the non-parametric estimate of the age-specific cumulative risk functions. We estimated life expectancy by numeric integration of the survival function, i.e. the complement of the cumulative-risk function, over the observed range of ages at mortality in the data. We obtained confidence intervals and standard error for parameter estimated by bootstrap sampling (500 replicates) of the WAS families in a way that automatically accounts for the correlation of the relatives within a family. For a global test of the difference in mortality risk between carriers and non-carriers, we compared their average life-expectancies based on estimates of the age-specific risk of mortality. Wald statistics were computed by parameter estimates divided by their bootstrap standard errors, and the associated p-values were reported.

### Mortality in the absence of all cancer

We also estimated mortality risk in the absence of any cancer diagnosis using the same method with follow-up censored at the time of cancer diagnosis at any site.

To examine possible impact of birth cohort effects in our analysis, we first performed tests for interactions in the Cox proportional hazard regression model to detect potential differences in the mortality risk between carriers and non-carriers by birth cohort. As no significant differences were found, we adjusted for the effect of birth cohort assuming the effect on mortality is the same for both carriers and non-carriers.

## Results

Participants who tested positive for one of the *BRCA* Ashkenazi founder mutations reported having a higher proportion of female siblings and children than did participants who tested negative. On average, mutation carriers and their relatives were born earlier than non-carriers and their relatives (Table 1). Twenty-seven percent of relatives of carriers were dead at the time of the study, compared with 25% of relatives of non-carriers. Ten percent of siblings of carriers were dead at the time of the study, compared with 16% of siblings of non-carriers. On the average, siblings of carriers were born four years later than siblings of non-carriers. Deceased parents and siblings of carriers had younger ages at death than parents and siblings of non-carriers.

**Table 1:** Descriptive statistics for participants and first-degree relatives from the Washington Ashkenazi Study.

	Participants			Parents of			Siblings of			Children of		
	Carriers (N = 120)	Non-carriers (N = 5167)	p-value^2^	Carriers (N = 232)	Non-carriers (N = 10146)	p-value^2^	Carriers (N = 178)	Non-carriers (N = 8185)	p-value^2^	Carriers (N = 184)	Non-carriers (N = 8599)	p-value^2^
% Male	25.83	29.86	0.34	50.00	49.96	0.99	45.51	51.25	0.13	45.11	51.05	0.11
Median year of birth	1948	1946	0.07	1917	1914	0.10	1950	1947	0.0008	1978	1972	0.0002
% Alive				40.95	47.00	0.07	89.89	84.23	0.04	98.37	98.24	0.90
Median age of death^1^				68	73	0.0006	51	58	0.86	22	10	0.87
Median year of death^1^				1979	1978	0.93	1984	1981	0.54	1989	1981	0.17

1 Among deceased relatives.

2 Chi-squared test for percentages or two-sample t-test for continuous variables.

**Figure 1:**
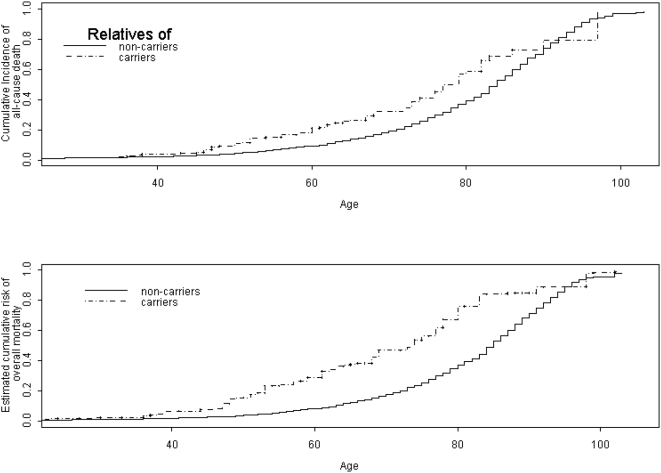
Cumulative incidence of all-cause mortality. Panel 1 shows the cumulative incidence of all-cause mortality among female first-degree relatives of participants in the Washington Ashkenazi Study. Panel 2 shows the corresponding kin-cohort estimate of cumulative mortality risk among female carriers and non-carriers of *BRCA1/2* Ashkenazi Jewish founder mutations.

Figure 1 shows the all-cause mortality for the cohort of female FDR of mutation carriers and non-carriers (panel 1) and the mortality risk in female carriers and non-carriers as estimated by the Chatterjee-Wacholder kin-cohort estimation method [16] (panel 2). The cumulative incidence of deaths among female FDR of carriers was higher than that for female FDR of non-carriers.

### Mortality in the absence of *BRCA*-related cancers

**Figure 2:**
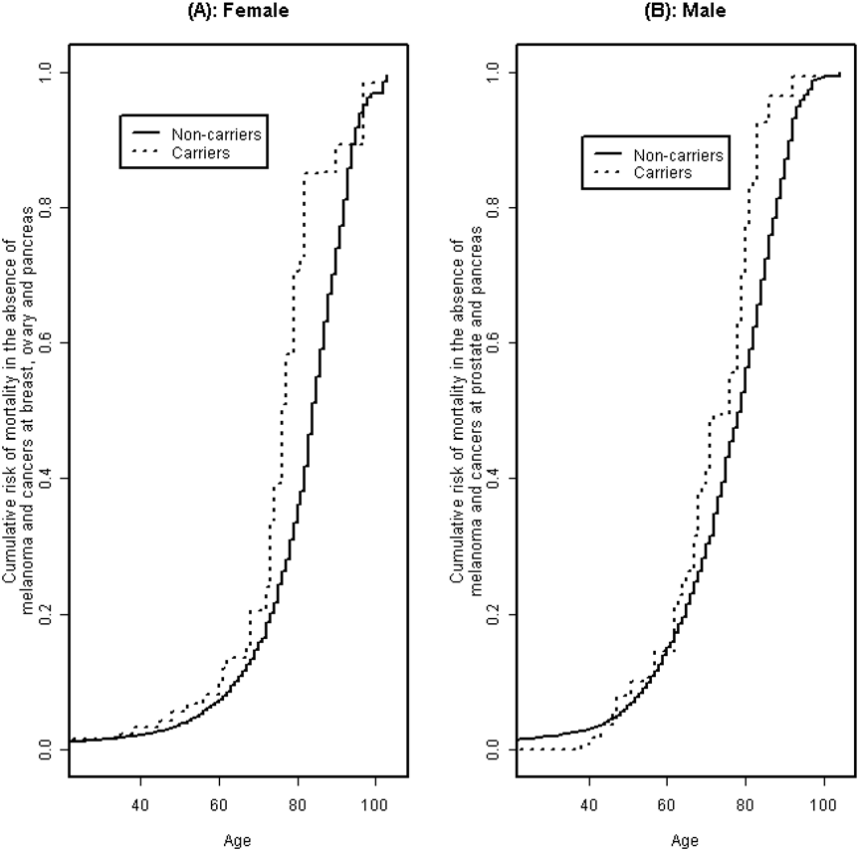
Cumulative incidence of mortality risk in the absence of established *BRCA* mutation-associated cancers. Estimated cumulative risk of mortality among females in the absence of breast, ovarian, pancreatic cancer, and melanoma is shown in panel A. Panel B shows the estimated cumulative risk of mortality among males in the absence of melanoma, prostate, and pancreatic cancer.

Table 2 shows the estimated mortality after excluding deaths and follow-up time in survivors of cancers of the breast, ovary, pancreas, or prostate, or melanoma by the modified kin-cohort method that accounts for dependent censoring. Mortality risk in female mutation carriers is elevated compared with female non-carriers in a number of age groups with the increase being more than twice and statistically significant at ages 71–80. The estimated cumulative mortality up to age 80, excluding time after breast, ovarian, pancreatic cancer and melanoma diagnosis, was 36% (95% CI: 34, 39) for non-carriers and 71% (95% CI: 47, 90) for carriers (Figure 2A). We further estimated that, in the absence of these cancers, the average life expectancy was 80.8 (95% CI: 80.4, 81.3) years for non-carriers and 75.0 (95% CI: 70.5, 79.4) years for carriers, or a difference of 6.8 (95% CI: 1.3, 10.5) years.

**Table 2:** Estimated hazard of mortality due to carrying a *BRCA* mutation in the absence of melanoma and cancer of the breast, ovary, pancreas, and prostate^1^.

Age	Female			Male		
	Non-carriers	Carriers	Hazard Ratio	Non-carrier	Carrier	Hazard Ratio
<50	.038 (.034, .042)	.056 (.018, .102)	1.46 (0.50, 2.82)	.066 (.061, .071)	.079 (.026, .142)	1.19 (0.38, 2.20)
51–60	.038 (.033, .044)	.047 (.000, .129)	1.26 (0.00, 3.42)	.092 (.084, .099)	.073 (.023, .152)	0.80 (0.25, 1.68)
61–70	.091 (.082, .101)	.118 (.021, .284)	1.29 (0.22, 3.07)	.179 (.168, .192)	.312 (.166, .451)	1.74 (0.89, 2.55)
71–80	.242 (.225, .260)	.630 (.334, .857)	2.60 (1.39, 3.64)	.374 (.353, .396)	.612 (.320, .825)	1.64 (0.85, 2.25)
81–90	.593 (.556, .629)	.634 (.143, .972)	1.07 (0.25, 1.65)	.704 (.668, .741)	.844 (.418, .980)	1.20 (0.58, 1.42)
91–100	.880 (.816, .936)	.853 (.030, .975)	0.97 (0.03, 1.08)	.947 (.874, .997)	.820 (.000, .100)	0.87 (0.00, 1.08)

1 Follow-up time and deaths were censored at first report of melanoma or cancer of the breast, ovary, pancreas, or prostate.

For men, we saw a similar but more modest effect of the mutations on mortality with censoring at time of melanoma, prostate, and pancreatic cancer diagnosis (Table 2 and Figure 2B). The estimated cumulative mortality risk until age 80 in the absence of these selected cancers was 56% (95% CI: 55, 58) for non-carriers and 77% (95% CI: 58, 90) for carriers. Finally, we estimated that, in the absence of melanoma and pancreatic and prostate cancer, the average life expectancy was 74.7 years (95% CI: 74.3, 75.1) for non-carriers and 71.0 years (95% CI: 68.1, 74.2) for carriers, or a difference of 3.7 (95% CI: 0.4, 6.8) years.

### Mortality in the absence of all cancer

**Figure 3:**
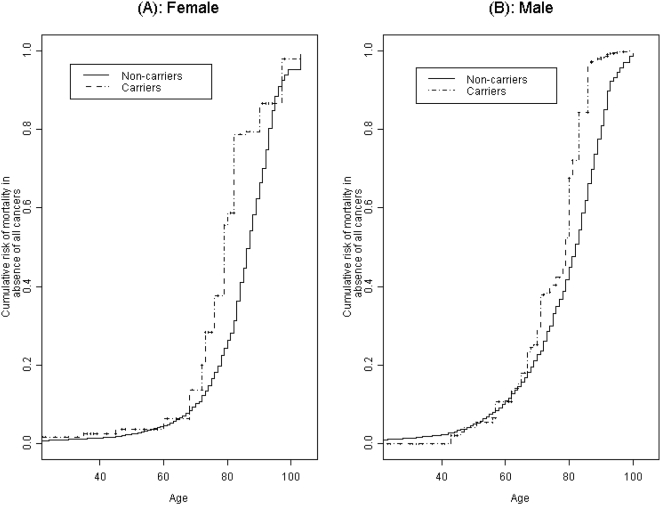
Estimated cumulative risk of mortality in the absence of any cancer. The figure shows the estimated cumulative risk of mortality in the absence of any cancer among females (panel A) and males (panel B).

Because cancers other than breast, ovary, pancreas, and prostate, and melanoma could be related to *BRCA1/2* mutations, we also estimated risks of mortality with censoring at time of diagnosis of any cancer (Table 3 and Figure 3). Among women, the mortality differences seen in Table 2 for the age group 61–70 persisted but at a reduced level. The estimated average life expectancy, in the absence of any cancers, was 5.7 years (95% CI = −0.1, 10.4) greater in female non-carrier than female carriers (83.8 (95% CI = 83.3, 84.3) vs. 78.1 (95% CI = 73.5, 83.9)). Among males, the estimated average life expectancy, in the absence of all cancers, was 78.0 years (95% CI = 77.6, 78.5) for non-carriers and 74.3 years (95% CI = 71.3, 78.1) for carriers – a difference of 3.7 years (95% CI = −0.4, 6.9). Although the difference in life-expectancies between carriers and non-carriers for females and males separately was only marginally significant at the 0.05 *α*-level, a test of association for the difference combining males and females based on Fisher's sum of chi-square statistics was significant with a p-value of 0.024.

**Table 3:** Estimated hazard of mortality due to carrying a *BRCA* mutation in the absence of any cancer^1^.

Age	Female			Male		
	Non-carriers	Carriers	Hazard Ratio	Non-carrier	Carrier	Hazard Ratio
<50	.026 (.023, .029)	.038 (.007, .074)	1.47 (0.28, 2.90)	.050 (.049, .055)	.042 (.008, .091)	0.85 (0.16, 1.87)
51–60	.020 (.016, .024)	.027 (.000, .098)	1.37 (0.00, 5.34)	.064 (.056, .070)	.067 (.009, .136)	1.05 (0.14, 2.22)
61–70	.059 (.052, .067)	.078 (.000, .205)	1.31 (0.00, 3.45)	.130 (.120, .142)	.209 (.083, .379)	1.60 (0.62, 2.91)
71–80	.181 (.163, .199)	.520 (.221, .802)	2.88 (1.16, 4.62)	.288 (.267, .307)	.540 (.245, .809)	1.88 (0.82, 2.87)
81–90	.543 (.502, .584)	.675 (.139, .957)	1.24 (0.28, 1.79)	.653 (.607, .689)	.946 (.249, .972)	1.45 (0.39, 1.55)
91–100	.852 (.780, .928)	.847 (.783, .979)	0.99 (0.91, 1.13)	.972 (.833, .997)	.972 (.000, .999)	1.00 (0.00, 1.01)

1 Follow-up time and deaths were censored at first report of any cancer.

We also estimated the overall mortality in the absence of any cancer for *BRCA1* and *BRCA2* mutation carriers separately. Among women, the difference in life expectancy between carriers and non-carriers was 4.2 years for *BRCA1* mutations and 5.5 years for *BRCA2* mutation. For men, the corresponding difference was 5.0 years for *BRCA1* mutation carriers and 2.7 years for *BRCA2* mutation carriers. None of these differences were statistically significant.

Estimates from sensitivity analyses showed consistent effects, though often without statistical significance in subgroups. When adjusted for birth cohort, the excess in mortality due to *BRCA1/2* mutations increased slightly for women. The overall pattern of increased estimated mortality risk for carriers was observed in all three generations, parents, siblings and children. We excluded follow-up information referring to time before 1975 to reduce possible bias that may arise due to deaths following unreported cancers that occurred in the distant past; following this exclusion, in the absence of all cancers, the mortality difference between carriers and non-carriers increased; with an estimated gap in life-expectancy of 10.3 years for women and 7.5 years for men.

## Discussion

In this study, we observed an overall association between *BRCA1/2* mutations and reduced life expectancy after excluding deaths following diagnosis of the cancers that have been shown to be related to these mutations. The effects of the mutations were mainly manifested through increased hazard of mortality between ages 61 and 80. Further, the reduction in estimated life expectancy persisted after excluding deaths following any cancer diagnosis.

To our knowledge, this is the first report to date addressing the affect of *BRCA* mutations on non-cancer mortality risk. It is commonly thought that overall life expectancy is reduced among *BRCA* mutation carriers; however, no accurate estimates of this reduction exist, and the contributing cause is thought to be mainly breast and ovarian cancer [14], [15]. Furthermore, *BRCA*-related breast cancer have been thought to be associated with a worse prognosis than sporadic cases; however, existing data are inconsistent, with several studies suggest that there is no difference in survival between patients with hereditary and patients with sporadic breast cancer, particularly among patients treated with chemotherapy [22]–[25]. *BRCA*-related ovarian cancer, on the other hand, has been shown to be associated with an improved survival compared with sporadic cases among patients of Jewish descent [26]–[28]. The difference in survival after a breast or ovarian cancer diagnosis among *BRCA* mutation carriers compared with sporadic cases cannot explain the findings of this report since a mortality difference between among mutation carriers and non-carriers persisted after excluding major *BRCA*-related cancers as well as all cancers.

*BRCA1/2* genes have been shown to play major role in maintaining genomic stability, but the exact mechanism remains unknown [13]. Premature aging with decreased life-span has been observed in animal studies using mice that were homozygous for a hypomorphic mutation *Brca1* allele and heterozygous for wild-type *p53* (*Brca^Δ11/ Δ 11^ p53* **^+/−^**) [29]; however, the mechanism by which *BRCA* genes exert their affect on mortality is not known. In vivo studies and mice model studies have suggested that *BRCA1/2* is involved in translation regulation and the Insulin-Like Growth factor signaling axis [30], [31], but whether these pathways are directly related to premature aging is not clear.

The major strength of our study is that it is based on a large community sample rather than on high-risk family registries or cancer case series. The retrospectively constructed time of follow-up for the relatives of the study participants gave us a unique opportunity to estimate age-related mortality risks associated with *BRCA1/2* mutations.

One limitation of our study is the potential bias due to under-reporting by WAS participants of cancer diagnoses in their relatives. The known high risk of certain cancers among carriers could lead to substantial overestimation of mortality risk seemingly from other causes if cancers were under-reported in this group. At the time this study was done, clinical testing for *BRCA1/2* was not available, and participants were not aware of their mutation status. Thus, we would not expect differential reporting due to genotype. However, even if the misreporting was non-differential between mutation carriers and non-carriers, the greater number of missed mutation-related cancers in mutation carriers could exaggerate the effects. Nevertheless, the mortality differences we observed for both women and men seem too great to be explained completely by deaths following unreported cancers. Moreover, the findings did not change significantly when the data was limited to relatively recent deaths or stratified by generations. This observation suggests that the apparent mortality difference was unlikely to be entirely due to under-reporting of cancers in relatives.

The major limitation of this report is that the WAS did not ask participants about non-cancer causes of death in relatives. Consequently, we cannot identify specific conditions that might explain the observed association between the three *BRCA1/2* Ashkenazi founder mutations and non-cancer mortality. We censored follow-up duration at the time of any cancer diagnosis, and used a statistical method [19] that ensures that the observed association between mutation status and mortality were not due to any reported cancers. Previously, we showed that in this study population, the overall mortality after breast or ovarian cancer diagnosis was similar in relatives of carriers and non-carriers [18]. Moreover, other studies of Ashkenazim have found no difference in overall survival between *BRCA* mutation carriers and non-carriers after a diagnosis of breast cancer [24], and have suggested a more favorable overall survival among women with *BRCA*-related ovarian cancer [27].

In conclusion, we observed an excess in non-cancer mortality associated with the founder *BRCA1/2* mutations among an Ashkenazi Jewish cohort, with a reduction in life expectancy of approximately 4–6 years. If real, this observed excess mortality risk in the absence of any cancer suggests that *BRCA1/2* mutations may exert biologic effects quite apart from their well-established influence on cancer risk. Theoretically, these mutations may either be associated with a small increase in risk of a variety of different diseases, or they may be associated with moderate increase in risk of a few major diseases. There are currently very little available data on the effects of *BRCA* mutations on either overall or cause-specific mortality, and there has been no study investigating the association between *BRCA1/2* mutations and non-malignant conditions. However, before speculating on specific mechanisms by which *BRCA1/2* mutations might contribute to non-cancer conditions, confirmatory studies are essential. Moreover, although the findings in our study are intriguing, it is not clear whether the same findings can be generalized to persons with *BRCA1/2* mutations not studied in the current cohort, or to other, non-Ashkenazi populations. Thus, additional studies, both among Ashkenazim and with other unselected *BRCA1/2* carrier populations, are needed to verify this finding. Identifying underlying mechanisms for effects of *BRCA1/2* on non-cancer mortality, if the finding is confirmed, could lead to a better understanding of the biological basis through which the *BRCA1/2* genes exert their impact on cancer risks as well as on other non-cancer conditions. Furthermore, insights into the mechanism of action of the *BRCA1/2* genes could in turn facilitate research efforts aimed at finding innovative preventive management options for *BRCA1/2* mutation carriers.
